# Impact of the SARS-CoV-2 Pandemic on the Prevalence and Incidence of Gastrointestinal Viruses in Children up to Five Years Old: a Retrospective Cohort Study

**DOI:** 10.1128/spectrum.02669-21

**Published:** 2022-05-31

**Authors:** Alfredo Maldonado-Barrueco, Julio García-Rodríguez, Jorge Yániz-Ramirez, Irene Serrano-Vaquero, Juan Carlos Parra-Alonso, Carlos Vega-Nieto, Guillermo Ruiz-Carrascoso

**Affiliations:** a Clinical Microbiology and Parasitology Department, Hospital Universitario La Paz, Madrid, Spain; b Faculty of Medicine, Universidad Autónoma de Madrid, Madrid, Spain; U.S. Food and Drug Administration

**Keywords:** SARS-CoV-2, adenoviruses, astrovirus, children, gastrointestinal viruses, noroviruses, oral vaccines, rotavirus, sapovirus

## Abstract

The aim is determining the impact of non-pharmaceutical measures (NPIs) against SARS-CoV-2 in the incidence and prevalence of gastrointestinal viruses (GV) in children. Demographic, analytical, and clinical data of children from which samples were received at the Hospital Universitario La Paz (Madrid, Spain) and that had a gastrointestinal infection with a positive sample through multiplex-PCR for GV were collected. The time periods included were prepandemic (P1): March 14, 2019 to March 14, 2020 and pandemic (P2): March 15, 2020 to March 15, 2021. The global prevalence, relative incidence (RI, per 1,000 admissions) and absolute incidence (AI, per 100,000 population) of GV were compared for both time periods. The prevalence of GV versus SARS-CoV-2 was determined for P2. Seven-hundred and 50 out of 2,547 children analyzed in P1 and 106 out of 1,368 in P2 were positive by PCR for GV (46.3% decrease in P2). Prevalence and RI of GV declined in P2, except for the RI of rotavirus. Adenovirus showed the largest decreased of prevalence and RI (100%), followed by sapovirus. Astrovirus reduction was less pronounced (3.1% versus 0.4%). Norovirus was the most frequent virus in both time periods and its prevalence and RI also decreased in P2 (15.2% versus 4.7% and 3.40 versus 1.74, respectively). Rotavirus had the smallest decrease in prevalence (2.6% versus 2.5%), and its RI increased during P2 from 0.7 to 0.93. After removing the rotavirus vaccine strains from the analysis, the prevalence and RI decreased during P2 (2.1% to 0.7% and 0.5 to 0.3, respectively). The AI decreased during P2 in all GV, and the prevalence of SARS-CoV-2 and GV was inversely proportional over time. Prevalence and incidence of GV have decreased during the pandemic, probably due to the implementation of NPIs against this virus and the reduction of health care attention to infections other than COVID-19. The differences in the decrease of prevalence and incidence for each virus may be explained by differences in the transmission and the resistance in the environment. Prevalence and RI of rotavirus might be biased since the PCR used detects both the infecting and the vaccine strains.

**IMPORTANCE** Our original article contains an analysis of the impact of the measures applied against SARS-CoV-2 on the prevalence and incidence of GV in children. The small number of studies published to date that analyze the impact of these measures individually on each of the GV makes our study of great interest at this time.

## INTRODUCTION

On March 14, 2020, the State of Alarm was declared in Spain due to the pandemic caused by SARS-CoV-2. The movement of all citizens of the country was restricted except for essential personnel. At that time, strict limits on home visits and socializing in public places were put in place. Sanitary hygiene measures, such as frequent hand washing, the use of hydroalcoholic gels, and universal mask wearing were made mandatory in all types of indoor and outdoor environments. Moreover, the maximum amount of persons allowed in closed spaces was reduced. In the case of children, face-to-face educational activities were suspended in all areas (including parks and other outdoor areas). Medical activity in the hospital and primary care given to these patients was greatly restricted due to the collapse caused by the avalanche of patients with COVID-19 symptoms.

These lockdown measures were shown to have a significant impact on the circulation of other non-SARS-CoV-2 respiratory viruses like influenza and respiratory syncytial virus (1, 2), but the data about the effect of these measures on the circulation of gastrointestinal viruses (GV) is scarce (3–5). Viral gastroenteritis represents 70% to 90% of all infectious gastroenteritis in children under 5 years old (6, 7). The symptoms are usually unspecified and, in most cases, the disease is self-limited and recovery happens in a few days. However, each year, viral gastroenteritis causes 200,000 childhood deaths around the world, and is mainly associated with the cold seasons (8). The transmission of GV can occur mainly via fecal-oral (food or water), vomitus, fomites or drops, and they are frequent agents of community and nosocomial outbreaks.

The objective of this study is to determine the impact of the SARS-CoV-2 pandemic on the prevalence and incidence of GV in children with samples handled at our hospital by comparing data obtained before and during the pandemic.

## RESULTS

The two periods of time analyzed were the prepandemic period (P1) from March 14, 2019 to March 14, 2020, and the pandemic period (P2) from March 15, 2020 to March 15, 2021. During P1, real-time PCR (rt-PCR) for the detection of GV was performed for 2,547 children up to 5 years old. During P2, this detection was performed for 1,368 children (i.e., 46.3% fewer patients analyzed in P2). Primary care, was greatly reduced due to the overload in the system caused by the care of COVID-19 patients during P2, partly justifying the drop in the number of PCR analyses for GV (a 41% decrease from 731 children in P1 to 432 in P2). During P1, the number of positive PCRs for any of the GV was 759 out of 2,547 children (29.8%) while in P2 it decreased to 106 out of 1,368 children (7.7%). The difference between both periods was statistically significant (*P *< 0.05) for all the months except for August and February (Table 1).

**TABLE 1:** Children up to 5 years old with PCR positive for gastrointestinal viruses and percentage of positivity (%)

March 14, 2019 to March 14, 2020 (*n* = 2,547)							March 15, 2020 to March 15, 2021 (*n* = 1,368)							
Month	Adenovirus	Astrovirus	Norovirus	Rotavirus	Sapovirus	TOTAL	Month	Adenovirus	Astrovirus	Norovirus	Rotavirus	Sapovirus	TOTAL	*P*-value
March	5 (3.2%)	5 (3.2%)	18 (11.5%)	23 (14.7%)	5 (3.2%)	56	March	0 (0%)	1 (2.4%)	0 (0%)	2 (4.8%)	1 (1.3%)	4	<0.05
April	6 (2.9%)	6 (2.9%)	22 (10.7%)	20 (9.7%)	8 (3.9%)	62	April	0 (0%)	0 (0%)	2 (3.2%)	2 (3.2%)	0 (0%)	4	<0.05
May	3 (1.5%)	2 (1%)	17 (8.7%)	6 (3.1%)	13 (6.6%)	41	May	0 (0%)	0 (0%)	0 (0%)	2 (1.9%)	0 (0%)	2	<0.05
June	2 (1.0%)	2 (1%)	25 (12.7%)	2 (1.0%)	10 (5.1%)	41	June	0 (0%)	0 (0%)	4 (3.3%)	1 (0.8%)	0 (0%)	5	<0.05
July	5 (2.3%)	1 (0.4%)	23 (10.5%)	2 (0.9%)	11 (5%)	42	July	0 (0%)	0 (0%)	2 (1.4%)	4 (2.8%)	0 (0%)	6	<0.05
August	1 (0.5%)	1 (0.5%)	8 (4.4%)	0 (0%)	5 (2.7%)	15	August	0 (0%)	2 (1.6%)	2 (1.6%)	3 (2.4%)	0 (0%)	7	0.76
September	3 (1.3%)	1 (0.4%)	44 (19.5%)	1 (0.4%)	11 (4.9%)	60	September	0 (0%)	0 (0%)	1 (0.9%)	3 (2.6%)	0 (0%)	4	<0.05
October	11 (3.4%)	2 (0.6%)	133 (41%)	0 (0.0%)	39 (12%)	185	October	0 (0%)	0 (0%)	3 (2.9%)	4 (3.9%)	0 (0%)	7	<0.05
November	14 (5.6%)	13 (5.2%)	45 (18.1%)	1 (0.4%)	40 (16%)	113	November	0 (0%)	0 (0%)	6 (4.9%)	2 (1.6%)	0 (0%)	8	<0.05
December	2 (1%)	19 (10%)	18 (9.5%)	0 (0.0%)	11 (5.8%)	50	December	0 (0%)	0 (0%)	13 (9.2%)	2 (1.4%)	0 (0%)	15	<0.05
January	1 (0.6%)	14 (8.4%)	15 (8.9%)	6 (3.6%)	7 (4.2%)	43	January	0 (0%)	2 (1.7%)	2 (1.7%)	6 (5.1%)	1 (0.8%)	11	<0.05
February	4 (2.3%)	11 (6.4%)	16 (9.3%)	3 (1.7%)	8 (4.7%)	42	February	0 (0%)	0 (0%)	10 (9.6%)	2 (1.9%)	1 (0.9%)	13	0.07
March	1 (1.5%)	3 (4.5%)	3 (4.5%)	2 (2.9%)	0 (0%)	9	March	0 (0%)	0 (0%)	19 (25%)	1 (1.3%)	0 (0%)	20	<0.05
TOTAL	58 (2.3%)	80 (3.1%)	387 (15.2%)	66 (2.6%)	168 (6.6%)	759	TOTAL	0 (0%)	5 (0.4%)	64 (4.7%)	34 (2.5%)	3 (0.2%)	106	<0.05

During P2, the prevalence of each virus tested for was lower, with the exception of rotavirus. In relation to P1, the highest prevalence of GV was during October 2019, and the lowest was in March 2020. In addition, during P2, the rate of positivity showed a valley in May 2020 and a peak in March 2021 (Table 1). In terms of individual viruses, the prevalence for adenovirus was reduced 100% (*P *< 0.05); from 2.3% in P1 to 0% in P2. The decrease in sapovirus prevalence was 96.9% (*P *< 0.05); from 6.6% in P1 to 0.2% in P2. For astrovirus, the prevalence decreased from 3.1% to 0.4% which implies a 87.1% decrease (*P *< 0.05). Norovirus (I and II genogroups) showed a 69.1% decrease (*P *< 0.05); from 15.2% in P1 to 4.7% in P2. Rotavirus showed a decrease of 3.8% (*P *= 0.8); from 2.6% in P1 to 2.5% in P2 (Table 1).

**FIG 1:**
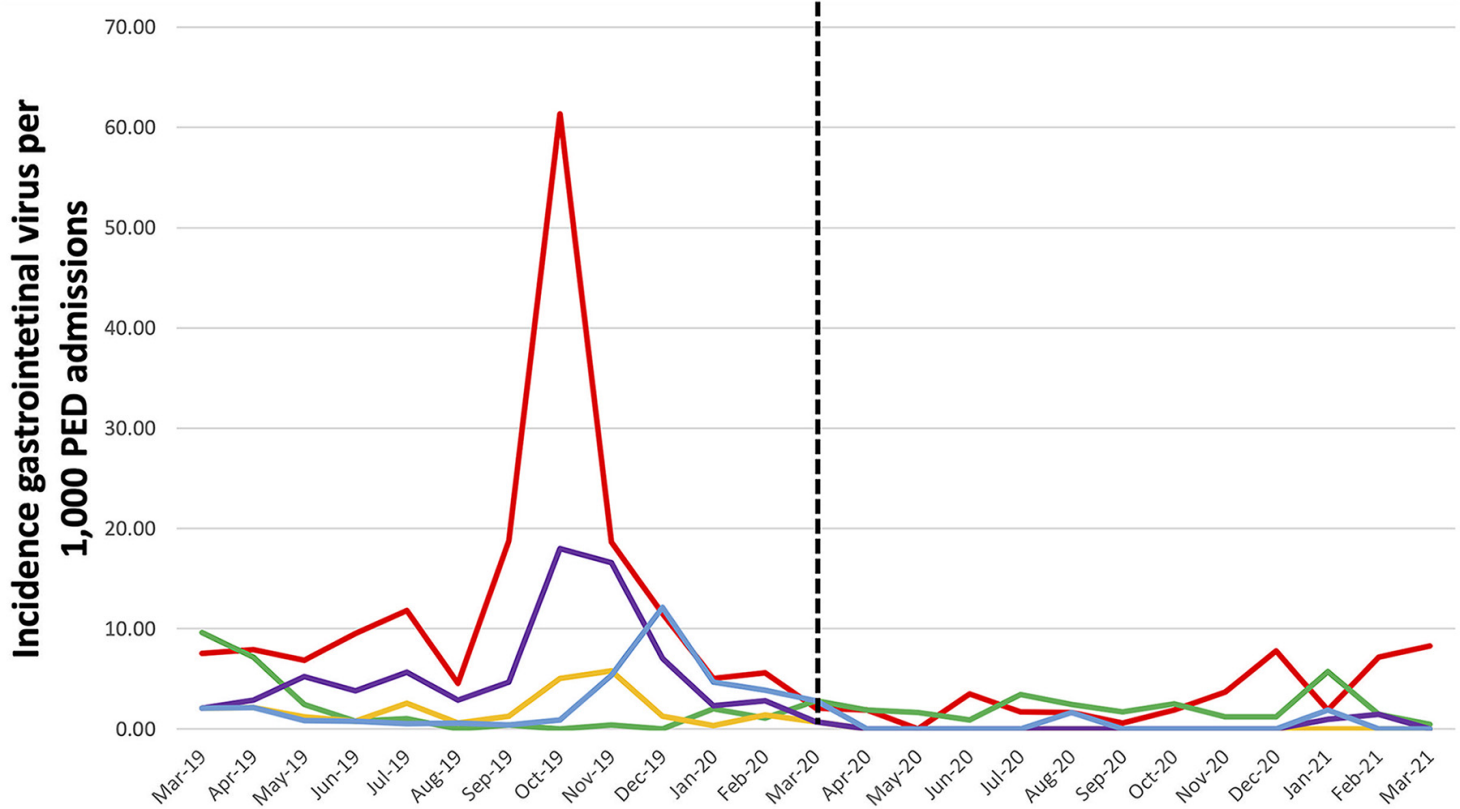
Rate of incidence on each of gastrointestinal virus per 1,000 pediatric emergency room (PED) admission versus months: adenovirus (orange line), astrovirus (blue line), norovirus (red line), rotavirus (green line), and sapovirus (purple line). Black slash line divide prepandemic period (P1) to pandemic period (P).

During P1, 29,734 children between 0 and 5 years old received medical attention at pediatric emergency department (PED). This number decreased during P2 to 58% (17,243 children) (Fig. 1). The major rate of incidence per 1,000 admissions during P1 was observed during the cold season (October 2019 to January 2020), but this incidence drastically decreased in P2. The relative incidence per 1,000 admissions and prevalence in the PED for each gastrointestinal virus in both periods decreased during P2, with the exception of rotavirus (Table 2). However, the rate of absolute incidence per 100,000 population in the PED decreased for all the GV, including rotavirus.

**TABLE 2:** Prevalence, relative incidence, and absolute incidence of gastrointestinal viruses in the pediatric emergency department

March 14, 2019 to March 14, 2020 (*n* = 29,734 admissions)				March 15, 2020 to March 15, 2021 (*n* = 17,243 admissions)				
Gastrointetinal viruses	Prevalence (%)	Relative incidence (x 1,000 admissions)	Absolute incidence (x 100,000 population)	Gastrointetinal viruses	Prevalence (%)	Relative incidence (x 1,000 admissions)	Absolute incidence (x 100,000 population)	*P-*value
Adenovirus	9.2	0.9	81.2	Adenovirus	0	0	0	<0.05
Astrovirus	2	0.2	18	Astrovirus	1.7	0.2	9.1	0.8
Norovirus	34.5	3.4	303.7	Norovirus	16.9	1.7	91.3	<0.05
Rotavirus	7.5	0.8	67.1	Rotavirus	9	0.9	48.7	0.6
Sapovirus	13.6	1.4	120.3	Sapovirus	0	0	0	<0.05

To analyze the impact of probable detection of the vaccine strains for the calculation of the prevalence and incidence of rotavirus, vaccination records of children up to 8 months old were analyzed. Among these children, rotavirus was positive by rt-PCR in 55: 25 cases in P1 and 30 in P2. In P1, 11 (84.6%) out of 13 infants with the vaccination status recorded were probably positive due to the detection of the vaccine strains, while 24 (92.3%) out of 26 infants with vaccination coverage were considered in this category during P2. Correcting the prevalence of rotavirus through removing the samples were possible vaccination strains were detected resulted in a decrease in prevalence from 2.6% to 2.1% in P1 and from 2.5% to 0.7% in P2. Similarly, correcting the relative incidence rates per 1,000 admissions of rotavirus in the PED resulted in a decrease from 0.8 to 0.5 in P1 and from 0.9 to 0.3 in P2. The absolute incidence rates per 100,000 population of rotavirus in the PED were higher in P1 than P2, regardless of the probable vaccine strain detection.

**FIG 2:**
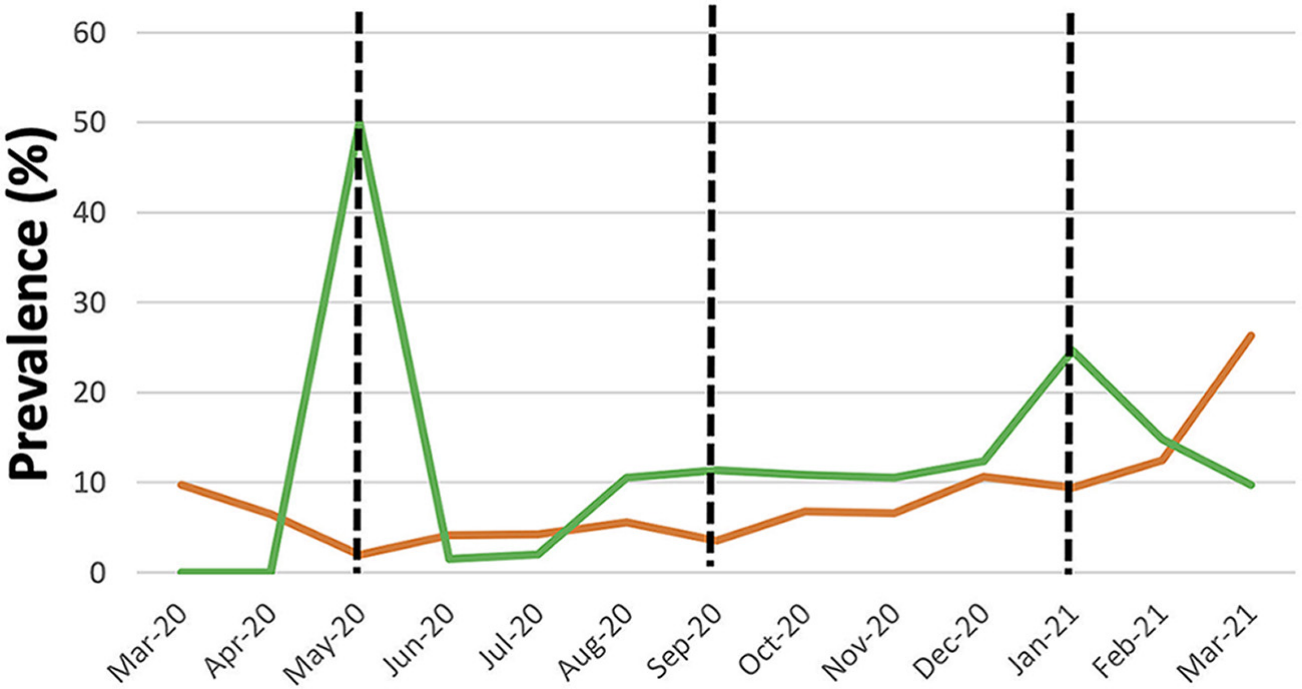
Prevalence of gastrointestinal virus PCR (green line), prevalence SARC-CoV-2 (brown line), versus months of pandemic period (P2). Black dash line divide the three pandemic peaks.

Since May 2020, SARS-CoV-2 testing through reverse transcription rt-PCR (RT-PCR) was initiated for children. Since then and up to March 15, 2021, a total of 12,504 SARS-CoV-2 RT-PCRs for children up to 5 years old were performed, of which 1,581 (12.6%) were positive. The highest prevalence was during the month of May 2020 and the lowest in June 2020 (Fig. 2). Three peaks during P2 were observed and paralleled the maximum peaks in the entire Spanish population. During these peaks, there was an inverse relation between positive GV and SARS-CoV-2 detection.

## DISCUSSION

In our study, the prevalence for GV in children up to 5 years old decreased almost 50% between prepandemic (P1) and pandemic (P2) periods. Health attention, mainly for outpatient children, was decrease due to the increase demand for care caused by COVID-19 during P2, partly justifying the drop in the number of PCR analyses for GV. The reduction of prevalence of GV happened especially during March, April, and May 2020 due to the strict confinement implemented by the national government, and the restrictions placed on the population, where only essential workers could leave home during working hours. On the other hand, a modest increase in the prevalence of the GV was observed at the end of P2 (December 2020 to March 2021) that could be explained by the de-escalation and relaxation of measures, and the returning to standard life norms such as reopening schools, kindergartens, daycare centers, playgrounds, and parks. In this period, the population retook travel and got back in touch with their friends and relatives. On the other hand, the noticeable decline in PED admissions could also be explained by the fact that viral gastroenteritis infections are usually self-limited and resolve in a few days (6). It is very likely that the families avoided taking the children to the PED during the pandemic if the child is not critically ill, resulting in far fewer cases during P2. Moreover, the implementation of non-pharmaceutical interventions (NPIs) with the objective to reduce the transmission of SARS-CoV-2 (such as wearing masks, surface disinfection, social distancing, hand hygiene, and restricted classroom-based education among children) could have also resulted in the decline of gastroenteritis cases in during P2. Along these lines, some reports did indeed show a decrease in the prevalence of other infectious viruses such as the respiratory syncytial virus and influenza virus, as a consequence of NPIs (1, 2), and especially among the children population (9, 10). Some authors also reported on the impact of SARS-CoV-2 on GV in Korea, Japan, Australia, and the United States, but they were mainly focused on norovirus (5, 11, 12).

Underreporting due to reduced health care utilization during pandemic might also have affected the results obtained during P2, but the analysis performed using data obtained from the PED should partially control for this since the patients receiving health care at the PED usually have more severe symptoms and are less likely to be biased by underreporting. Furthermore, the incidence analysis was performed in the PED because a great proportion (only surpassed by samples from outpatient clinics) of stool samples received during P1 and P2 come from this department. Furthermore, the GV detected in patients attended at PED represent more accurately the proportion of viruses circulating in the community rather than in other inpatient departments, where only the proportion of pediatric patients that they need hospitalization is represented and where outbreaks by GV (norovirus, rotavirus) can sometimes occur.

In terms of the prevalence and incidence for each virus included in this study, a dramatic decrease was observed for adenovirus. This may be due to the fact that the enteric adenovirus (serotypes 40/41) can be transmitted by inhalation of aerosolized drops, in addition to the frequent fecal-oral route (13). The application of NPIs could have interfered with this transmission route and might have led to this great decrease in prevalence and incidence. Norovirus was the most frequent gastrointestinal virus in both periods, but showed a marked decrease in prevalence and incidence during P2. This goes in accordance with the literature where norovirus is the most common viral cause of gastroenteritis (14), and might be due to the small viral load required to produce an infection, as well as the more extensive measures needed for eradication and the need of use of disinfectants that containing chlorine (15). Moreover, non-enveloped viruses (including norovirus) are not likely to be inactivated through the use of alcohol-based hand sanitizers (16) that were extensively used during P2. Vomitus, the production of viral aerosols and airborne transmission following vomiting have also been suggested (14, 17, 18) as an important focus of norovirus infection. Moreover, according with some authors norovirus have been isolated in nasopharyngeal specimens (13, 19, 20) although airborne transmission has not yet been demonstrated. The closing of classrooms and playgrounds, which reduced child-to-child contact, could be responsible for the decrease of norovirus cases (12, 21). The decrease in incidence and prevalence of norovirus during the COVID-19 pandemic was similar to reports from other countries like England, United States, Australia, or China (4, 5, 12, 22). However, some studies showed that this decrease does not seem to be explained by neither seasonality nor underreporting (12, 22). In our study, there was a slight increase in the incidence and prevalence of norovirus at the end of P2. This increase might be associated with the relaxation of NPI measures, or as a result of a new immune-escaped strain of norovirus that periodically emerge (4, 5). It would be interesting to track the prevalence and incidence of norovirus infections, as well as the other viruses included in this study, during a post-NPI period in order to determine whether they could return to prepandemic levels (12).

For astrovirus, the decrease in prevalence and incidence is mainly attributed to their transmission among children being closely related to health care settings and school environments (23, 24). The reduction of exposure to both these settings during P2 supports this decrease. As for sapovirus, the special care taken for infants in the age group that this virus usually affects (0 to 2 years old) (25, 26) in terms of restricting mobility and social contacts could explain the decrease in its prevalence and incidence.

Rotavirus slightly decreased in prevalence during P2 (Table 1) in a manner that is comparable to another study (11). Remarkably, the relative incidence per 1,000 admissions of rotavirus at the PED continues slightly increased in during P2. However, these results are biased by the decrease in the number of PED admissions in P2 in relation with P1. Moreover, almost 80% of the children under 8 months old with a positive rt-PCR for rotavirus have been vaccinated during the previous 3 months before. This could have a drastic effect on the data analyzed in our study and is one of its limitations. Another limitation is the fact that it was not possible to sequence the viral strains in order to differentiate between the vaccine strains and the infecting ones. One last limitation is that the vaccination data during P1 was not included because vaccination for this virus is not mandatory and there are no databases holding this information for all the patients included in our study. Nevertheless, upon adjusting the incidence of rotavirus at the PED per 100,000 population, a decrease during P2 was observed (73%), similarly to other the other viruses.

When the prevalence of SARS-CoV-2 and GV was compared one with the other, an inverse relationship between the GV and the three peaks of SARS-CoV-2 showed in Fig. 2 was observed. This could be explained by the increase in the application of NPI measures and restrictions during the SARS-CoV-2 peaks. The gradual increase in the prevalence of GV from September 2020 to March 2021 could be caused by the de-escalation and relaxation of NPIs measures, in addition to the expected increment of gastrointestinal virus infections during the winter season.

In conclusion, because of the SARS-CoV-2 pandemic, there has been a marked decrease in global prevalence and incidence of GV in our setting. The application of NPIs measures could justify this decrease, especially during peak transmission waves of SARS-CoV-2. However, not all measures to reduce transmission would be helpful to children (i.e., school closures) and future research should examine specific interventions that are likely to result in largest reductions in transmission without adversely affecting the social development of children.

## MATERIALS AND METHODS

### Population data analysis.

The study was performed at the Hospital Universitario La Paz (HULP), a 1,300 bed tertiary care hospital in Madrid (Spain). This hospital provides health care to 531,371 inhabitants and serves as a referral hospital for 23 outpatient clinics. Moreover, it receives stool samples for the detection of GV from hospitalized children, PED and outpatient children up to 5 years old. In a retrospective observational cohort study design, we collected the demographic, analytical and clinical data of children up to 5 years old with viral gastroenteritis (as confirmed by PCR) from the hospital database and laboratory informatics systems. Ethical approval for this study was obtained by the local ethics committee (PI-4745).

Only the first sample of each patient was registered in order to obtain a more representative data set. The exception to that rule was patients that had an infection during P1 and P2, and patients that had two different infections caused by two different viruses (even if they were within the same time period). In order to compare the relationship between GV and detection of SARS-CoV-2, we also analyzed the number of positive SARS-CoV-2 samples through PCR in children up to 5 years old. With the objective of comparing the prevalence and incidence of GV circulating in our population during both periods, the number of children up to 5 years old admitted to PED in P1 and P2 was recorded. The prevalence of each gastrointestinal virus, incidence per 1,000 admissions in PED (relative incidence), and the rate of incidence per 100,000 population (absolute incidence) were then calculated.

In the case that the PCR was positive for rotavirus, we reviewed the rotavirus vaccination dates in order to determine the possible detection of a rotavirus vaccine strain (two or three doses, depending on the type of vaccine: Rotarix or Rotateq). The Rotavirus vaccination is not mandatory in Spain and it is not included in the vaccination calendar of all Spanish provinces. For this, the vaccination data had to be collected from the medical records or from the local public health database, when available. Children with a positive PCR for rotavirus within 3 months after vaccination were considered as probable vaccine strain (27–29).

Fisher’s exact test was used for the comparisons between proportions. A *P*-value of less than 0.05 (*P* < 0.05) was considered as statistically significant.

### Microbiologic analysis.

The request of stool sample testing for children admitted at HULP or the PED is based on the following clinical findings: refusal to feed in infants, refusal to eat or drink anything for more than 8 h in children, moderate to severe dehydration, severe abdominal pain, lethargy or decreased responsiveness, and/or repeated vomiting. In the outpatient settings, the request of stool samples for microbiological testing is requested at the discretion of the health care provider. Macroscopic examination of the stool samples was performed upon receipt of the sample in the Microbiology Department. Only samples with a Bristol Stool Form Scale score of ≥5 were submitted for microbiological testing (30).

The diagnoses of viral gastroenteritis was carried out by rt-PCR test with the Allplex GI-Virus Assay (Seegene), in combination with automated DNA extraction and PCR setup using a Microlab STARlet Liquid Handling robot (Hamilton), according to the manufacturers’ instructions. The rt-PCR assay includes the following six viral targets: adenovirus, astrovirus, norovirus genogroup I (NoV-GI), norovirus genogroup II (NoV-GII), rotavirus, and sapovirus. SARS-CoV-2 detection was performed by RT-PCR from nasopharyngeal swabs with different PCR assays depending on the stock availability or clinical priority. The kits that were used were the TaqMan 2019-nCoV assay kit v1 (ThermoFisher), SARS-CoV-2 real-time PCR kit (Vircell), Allple 2019-nCoV Assay (Seegene), or Xpert Xpress SARS-CoV-2 (Cepheid).
